# Determinants of effective vaccine coverage in low and middle-income countries: a systematic review and interpretive synthesis

**DOI:** 10.1186/s12913-017-2626-0

**Published:** 2017-09-26

**Authors:** David E. Phillips, Joseph L. Dieleman, Stephen S. Lim, Jessica Shearer

**Affiliations:** 1 Institute for Health Metrics and Evaluation, University of Washington, Seattle, USA; 2 PATH, Seattle, USA

## Abstract

**Background:**

Many children in low and middle-income countries remain unvaccinated, and vaccines do not always produce immunity. Extensive research has sought to understand why, but most studies have been limited in breadth and depth. This study documents existing evidence on determinants of vaccination and immunization and presents a conceptual framework of determinants.

**Methods:**

We used systematic review, content analysis, thematic analysis and interpretive synthesis to document and analyze the existing evidence on determinants of childhood vaccination and immunization.

**Results:**

We documented 1609 articles, including content analysis of 78 articles. Three major thematic models were described in the context of one another. Interpretive synthesis identified similarities and differences between studies, resulting in a conceptual framework with three principal vaccine utilization determinants: 1) Intent to Vaccinate, 2) Community Access and 3) Health Facility Readiness.

**Conclusion:**

This study presents the most comprehensive systematic review of vaccine determinants to date. The conceptual framework represents a synthesis of multiple existing frameworks, is applicable in low and middle-income countries, and is quantitatively testable. Future researchers can use these results to develop competing conceptual frameworks, or to analyze data in a theoretically-grounded way. This review enables better research in the future, further understanding of immunization determinants, and greater progress against vaccine preventable diseases around the world.

**Electronic supplementary material:**

The online version of this article (10.1186/s12913-017-2626-0) contains supplementary material, which is available to authorized users.

## Background

Childhood vaccinations are among the most effective and cost effective public health interventions available [1, 2]. Yet, vaccine-preventable diseases caused approximately 14% of disability-adjusted life years (DALYs) in 2015 globally, and 15% of DALYs in Sub-Saharan Africa [3].

Many children remain unimmunized. Global coverage of diphtheria-tetanus-pertussis (DTP3) vaccination ranges from 75% in Africa to 96% in Europe [4]. Despite progress, socio-economic inequalities persist as well [5]. Furthermore, vaccines do not always provide immunity. For example, vaccination against measles has been estimated to be 85% effective in the United States and 66% effective in Mozambique [6, 7].

Extensive research efforts have been focused to understanding why vaccine coverage (vaccination) remains low, and why vaccines sometimes fail to produce immunity (immunization). Quantitative studies have sought to measure determinants using survey and other data. Systematic reviews have been published on both vaccination and immunization determinants in order to gather studies together [8–14]. Some have taken additional steps to develop a conceptual framework, i.e. general model for thinking about how determinants interoperate [8, 11]. Others assess health systems, describing bottlenecks and constraints to successful vaccine delivery from the supply-side, for example by applying the World Health Organization’s (WHO) Health Systems Building Blocks framework to new vaccine introductions [15–17].

Most studies and systematic reviews have been limited in breadth and depth however. Other authors have noted that studies seeking to directly measure determinants are rarely based on a grounded theoretical model such as a conceptual framework from systematic review [11, 18]. Systematic reviews focusing on vaccine coverage have only covered a subset of the published research [8, 11]. Reviews on vaccine effectiveness have not offered a complete account of potential determinants; they instead focus on a few important factors such as cold storage or administration [12–14]. Synthesis of a wide body of literature also faces challenges of combining heterogeneous study designs [19]. Previously proposed conceptual frameworks are often untestable in a quantitative sense, and therefore may have limited utility to independent researchers with new data to analyze [8, 11]. Most conceptual frameworks rely on narrative description of determinants more than explicit depictions of the pathways and interactions through which they are thought to influence immunization [8–14].

Public health practitioners and vaccine researchers would benefit from a systematic review and synthesis of determinants research. A complete listing of previous studies would be valuable as a standalone resource, since one of the most challenging aspects of developing a conceptual framework is initially amassing all available information for synthesis. Efforts to make sense of the literature could yield useful hypotheses, even competing hypotheses, about how different determinants lead to immunization. Future studies could collect data to measure the hypothesized determinants, and model them to quantify their impact in a more grounded way. Interventions could leverage such information to target determinants of greater need and evaluate programs with outcomes that are fit for purpose to enhance their efforts to improve immunization around the world.

The objective of this study is to generate a conceptual framework of the determinants of effective coverage of childhood vaccines. This will summarize, as succinctly as possible, determinants of vaccine coverage, the determinants of vaccine effectiveness, and relationships between them, and represent them in the form of a testable hypothesis. Special emphasis will be placed on including determinants that are relevant in low and middle-income countries (LMICs). We accomplish the study objective by conducting a systematic review of reasons for non-vaccination and reasons for vaccine failure. We perform three qualitative analyses on the information in the review, using critical interpretive synthesis defined in Dixon-Woods et al. 2006 [20].

## Methods

### Protocol, registration and preliminary review

A pre-defined protocol was followed, detailed in Additional file 1. This protocol was unregistered. A preliminary review was first conducted to inform future searches and analyses. This was carried out in an unstructured manner, iteratively relying on Google Scholar search results, backward/forward citation searches, examination of highly-cited articles and expert input. Citation network diagrams were generated as an aid to locate works that web searches did not immediately uncover, but which other researchers often reference. Further details can be found in Additional file 1.

### Eligibility criteria

Eligibility criteria were established in advance, using information from the preliminary review as a guide. Any English-language article that supplied evidence for at least one determinant of vaccine coverage or effectiveness was eligible for inclusion. Articles were excluded if they specifically pertained to any of the following topics without meeting the inclusion criterion: adult vaccines besides HPV, animal vaccines, levels and trends of coverage, consequences of utilization, health impact, highly-specific subpopulations (e.g. travelers, HIV-positive populations), vaccine effectiveness/efficacy/safety, disease treatment, pathobiology, future vaccines, cost effectiveness, general health care utilization, impact of a single intervention, vaccine manufacturing, promotion of a particular vaccine technology.

### Search

A set of 112 potential search terms (mostly various synonyms) was developed based on the preliminary review, these terms are listed in Additional file 2. The search strategy was as follows:

1. Order the potential searches according to their expected propensity to return unique and relevant results
2. Enter the first search term into Google Scholar and review the first 500 titles, adding eligible citations to a database
3. Enter the next search term into Google Scholar, again reviewing the first 500 titles for inclusion
4. Count the proportion of new and duplicate articles identified by the present search
5. Repeat steps 3 and 4 until the duplication percentage exceeds 33% for three consecutive searches
6. Conduct two PubMed searches with multiple MeSH terms and screen results in their entirety.

By this procedure, 14 Google Scholar and 2 PubMed searches were conducted. In addition, six special databases were screened in their entirety with the keywords “Vaccine” and “Immunization”. These databases were HealthSystemsEvidence.org, Cochrane Library, Journal of Systematic Reviews, Agency for HealthCare Research and Quality, Centre for Reviews and Dissemination and EPPI Centre [21–26]. Nine existing systematic reviews were discovered through this process, and their compete citation lists were also screened [9, 10, 13, 27–32].

### Study selection

All eligible articles and documents were catalogued in a citation database and systematically evaluated for relevance to the present study’s objectives. The objective of this stage was to organize the search results so that they could be analyzed in descending order of relevance.

Articles and documents were assigned a subjective relevance score (on a continuous scale ranging from 0 – irrelevant to the present study, 1 – exactly on-topic) based on a set of five criteria, listed in Table 1. Relevance was determined using the title and abstract. An assessment by the reviewer was used to judge relevance across the five criteria. Although some criteria could be objectively assessed (for example “comprehensiveness” was assessed based on whether the authors of the study claimed to have explored an exhaustive list of determinants, or only focused on a subset), some degree of subjectivity was required on the part of the reviewer to assign relevance scores. Articles were assigned higher scores if all five criteria were aligned with the study’s objectives, and lower scores if only some of the criteria were aligned with the study’s objectives. 78 articles were determined to be highly-relevant and were selected for analysis.

**Table 1:** Systematic review criteria

Inclusion	Exclusion	Relevance	Extraction
Supplies evidence for at least one determinant	Adult vaccines besides HPV	Comprehensiveness	Country/region
	Animal vaccines	Emphasis	Review
	Levels and trends of coverage	Geography	Study design
English language	Consequences of utilization	Novelty	Study population
	Health impact	Outcomes	Related studies
	Highly-specific subpopulations		Antigen(s)
	Vaccine effectiveness/efficacy/safety		Outcome(s)
	Disease treatment		Determinant(s)
	Pathobiology		Exhaustive
	Future vaccines		Proximity
	Cost effectiveness		Pathway(s)
	General health care utilization		Effect size(s)
	Impact of a single intervention		Theme(s)
	Vaccine manufacturing		Thematic excerpts
	Promotion of a particular vaccine technology		

### Data collection process

Starting with the most relevant articles, the information therein was systematically extracted and stored in a database. The objectives of this stage were to find a subset of articles large enough to perform content analysis, thematic analysis and interpretive synthesis (see subsequent sections), and to itemize and understand the content of that subset of articles.

### Data items

Fourteen variables were extracted from each study in selected set. Variables included study characteristics and content of study results and are listed in Table 1. A color-coding system was used, and all coded documents were stored digitally to ensure transparency and consistency between extractions. Data were extracted from the 78 selected articles in descending order of relevance.

### Risk of bias

Risk of bias was assessed at the study-level. The primary characteristics assessed were comprehensiveness of determinants explored, emphasis of the study (whether determinants were the primary purpose of the study) and geography (subnational, national, sub-population). Bias was minimized by excluding non-comprehensive studies or studies which focused on specific sub-populations from the highly-relevant set of 78 articles. Because this study was primarily focused on understanding which determinants are represented in the literature (and not the strengths of their correlations), it was not deemed necessary to assess risk of bias in study results, for example due to selection, attrition, or detection.

### Summary measures and content analysis

The aims of this study were primarily qualitative in nature. Hence summary measures of study results were limited to the descriptions and lists of determinants provided by the authors.

Content analysis was performed to characterize the itemized determinants from data extraction [19, 33]. The objective of this stage was to document and organize the data, specifically focusing on the determinants and pathways identified in the literature.

All discrete determinants mentioned in the extracted data were listed and systematically examined. This was done by maintaining a running list of determinants, resulting in concurrent, rather than sequential content analysis and data extraction. The list of determinants, as well as the text from which they were extracted, was repeatedly revisited to understand the context in which those determinants were described. Synonymous determinants and determinants with negligible conceptual differences (e.g. “not enough time” vs “too busy”) were condensed into a common set of terms. Finally, a frequency table of determinants was created, and related pathways were explored, starting with the most frequently-cited determinants.

The output from this stage was represented in a path diagram intended to represent the universe of determinants, according to the literature.

### Synthesis of results

In addition to content analysis, two qualitative methods were employed to synthesize across studies: Thematic Analysis and Critical Interpretive Synthesis.

### Thematic analysis

Thematic analysis was carried out to document the broader qualitative groups (i.e. themes) within which determinants are hypothesized to reside [19, 33]. The objectives of this stage were to describe the ways in which authors most commonly categorize determinants, and to bring together similar categorizations into a small number (three to five) of broad frameworks, termed thematic models.

Thematic analysis was accomplished by relying on the author descriptions. The categories they used to group determinants were listed along with excerpts of text in which they were described. Like content analysis, this step was conducted in an iterative fashion, seeking patterns between studies. It was anticipated that some studies would cite a more generalized sociological or health system theory as the source for their themes. In the event that multiple studies cited the same generalized theory those citations were also examined and included in the thematic analysis.

### Critical interpretive synthesis

The third analysis was interpretive synthesis of the literature, following guidelines from Dixon-Woods et al. 2006 [20]. This stage entailed critical analysis of the determinants identified through content analysis and categorizations identified through thematic analysis. The objective of this phase was to formulate a conceptual framework that draws from other researchers’ frameworks, and represent this framework in the form of a testable hypothesis. Critical interpretive synthesis was used in addition to thematic analysis in order to draw deeper understanding of the themes identified, and analyze the ways in which they were hypothesized to fit together. This approach was used because it details suitable a process for accomplishing the goal of generating a testable hypothesis [20].

Key methods in interpretive synthesis are known as reciprocal translational analysis (RTA), refutational synthesis and lines-of-argument synthesis (LOA), and are adapted from meta- ethnographical research [20, 34, 35]. RTA expands on the processes already described in the previous two sections (Thematic Analysis and Content Analysis). In short, RTA involves identifying broad concepts (themes) reported in each study, and identifying similarities between them. Refutational synthesis involves the opposite process; contradictions between studies (in terms of themes or descriptions of themes) are explored. This process lends insight to a conceptual framework by elucidating the gaps and discrepancies between studies in such a way that produces novel perspectives on common concepts. The final method (LOA) involves the most interpretive input on the part of the researcher. LOA entails integrating strongly-supported factors across studies and attempting to form a synthesizing argument, or a description of how they fit together, that is both succinct and understandable to commonplace audiences. The synthesizing argument often includes the generation of synthetic constructs, sometimes referred to as latent variables.

Information for interpretive synthesis came from multiple sources. Other studies which offered their own conceptual framework were used, as well as lessons from Thematic Analysis described above. In addition, broader theories which have been applied to describe health service utilization and health system strength were used [15, 36].

The ultimate testable hypothesis developed in this stage was also formulated through an iterative process, repeatedly revisiting previously-documented themes as new studies were examined (over the period of the review, see below) for further comparisons and contrasts. This stage also relied heavily on broader sociological and health systems theories to offer formalized and grounded structure [15, 36].

### Additional analyses

Besides those previously described, no additional analyses were performed.

## Results

### Study selection

A total of 35 web-searches were performed from February 2015 to May 2015, including Google Scholar (14 searches), PubMed (2 searches) special databases (9 searches) and backward citations (10 articles). In total, 9041 titles were examined for inclusion. Of those, 1621 eligible articles were identified, 12 studies were re-publications of another article (with a different name or publisher) and were excluded.

**Fig. 1:**
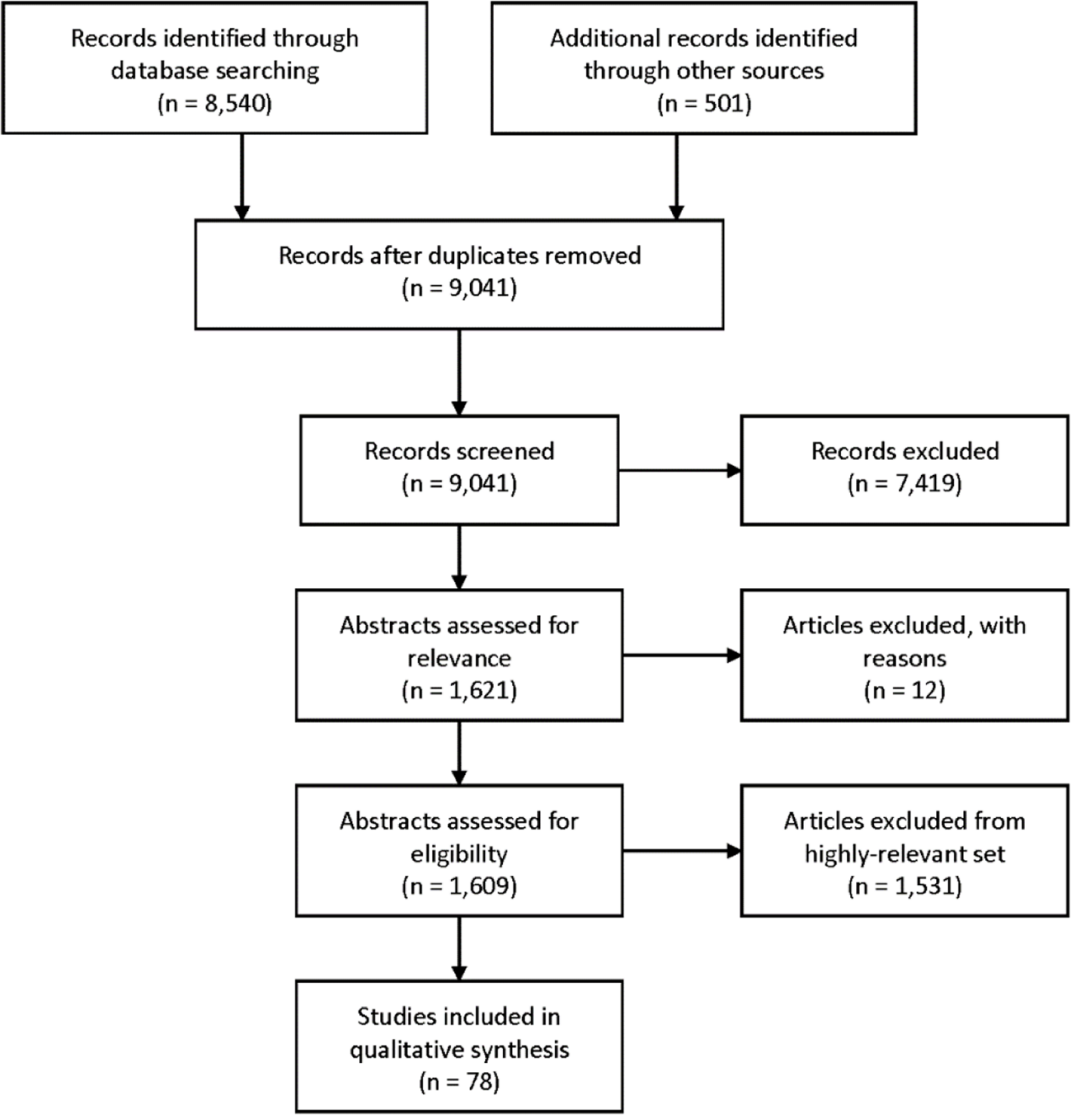
Flow diagram of search and selection. The systematic review assessed 1621 articles for their degree of relevance, and included the 78 most-relevant articles in qualitative synthesis

All 1609 included articles were assessed for relevance based on title and abstract, resulting in 78 highly relevant articles (relevance score > 0.9), 389 moderately relevant articles (relevance score > 0.8 and <0.9) and 1142 less-relevant articles (relevance score < 0.8). The complete list of 1609 citations can be found in Additional file 3, organized by relevance. Figure 1 displays a flow diagram of the search and selection.

### Study characteristics and content analysis

**Fig. 2:**
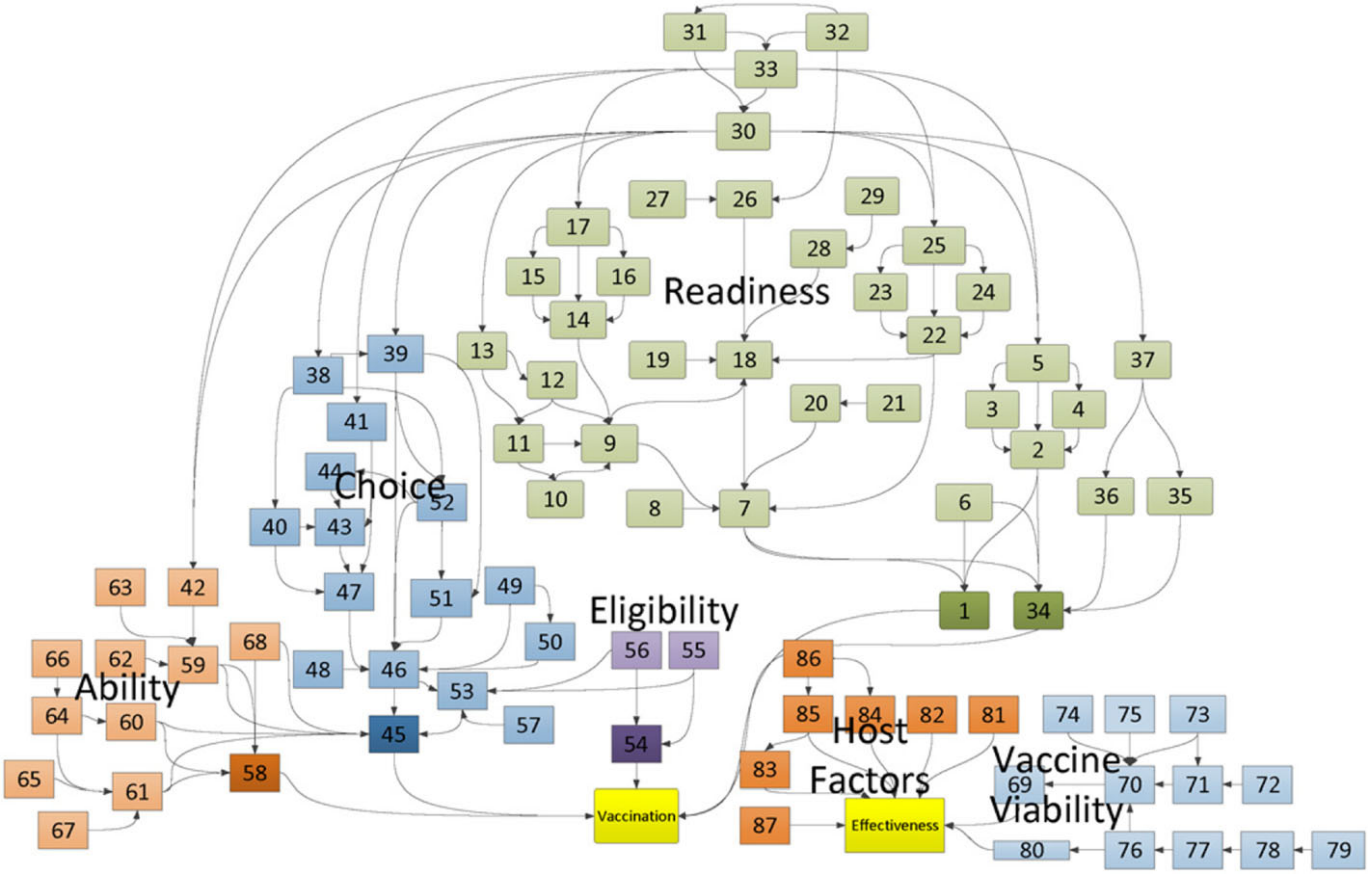
Network of determinants to vaccine effective coverage. This figure displays the full picture of determinants to vaccination and immunity. For details on individual determinants, refer to Table 2 and Table 3

Content was extracted from 78 articles, the characteristics of which are detailed in Additional file 3. A wide array of determinants was documented among the extracted articles, totaling 638 uniquely-worded factors such as travel time to health facility, various perceptions about vaccines, stock-outs at health facilities and others. By iteratively revisiting the list of determinants and examining the context in which they were described, a condensed framework of 69 coverage determinants and 20 effectiveness determinants was developed. By examining the described pathways between determinants, 115 pathways of influence were identified, forming a comprehensive network of vaccine coverage. An additional 22 pathways of influence were identified for vaccine effectiveness. The complete network of effective coverage determinants are listed in Tables 2 and 3, and displayed visually in Fig. 2.

**Table 2:** List of determinants to vaccine coverage

Theme	Determinant	Description	Relationship	Evidence	Label in Fig. 2
Facility Readiness	Facility Readiness (Routine)	Health facility is ready to vaccinate children	Directly impacts Utilization	[44]	1
	Service HR/Mgmt	Adequate health facility staff to meet demand	Directly impacts Facility Readiness	[11, 18, 31, 43–52]	2
	Training	Adequate health facility staff training	Moderates Service HR/Mgmt	[18, 27, 44, 49, 50, 51–58]	3
	Performance Assessment	Procedures for facility staff performance assessment	Moderates Service HR/Mgmt	[11, 18, 54, 59, 60]	4
	Disbursement/Fund Mgmt	Adequate resources and management for health facility staff	Directly impacts Service HR/Mgmt, Training, Performance Assessment	[18, 31, 44, 49]	5
	Waste Disposal	Procedures and equipment for vaccine waste	Directly impacts Facility Readiness	[36, 54]	6
	Facility Vaccine Supply	Health facility has vaccines to administer	Directly impacts Facility Readiness	[44, 48, 49–52, 55–58, 60, 61]	7
	Facility Storage Capacity	Health facility has sufficient capacity in refrigerators and freezers to store vaccines	Moderates Facility Vaccine Supply	[44, 50, 52, 61–64]	8
	Stock/Flow Awareness	Staff members know the status of vaccine inventory	Moderates Facility Vaccine Supply	[50, 52]	9
	Consumption Forecasting	Appropriate anticipation of future demand	Directly impacts Stock/Flow Awareness	[18, 47]	10
	Transaction Visibility	Adequate resources dedicated to supply chain transparency	Directly impacts Stock/Flow Awareness	[50, 52]	11
	Transaction Reporting	Adequate resources dedicated to communication of transactions	Directly impacts Stock/Flow Awareness	[50, 52]	12
	Supply LMIS	Logistics management information system for tracking supply	Moderates Transaction Reporting, Transaction Visibility	[50, 52]	13
	Inventory HR/Mgmt	Adequate resources dedicated to inventory management	Directly impacts Stock/Flow Awareness	[5, 18, 50])	14
	Training	Adequate inventory management training	Moderates Inventory HR/Mgmt	[18, 27, 44, 49, 50, 51–58]	15
	Performance Assessment	Procedures for inventory management performance assessment	Moderates Inventory HR/Mgmt	[11, 18, 54, 59, 60]	16
	Disbursement/Fund Mgmt	Adequate resources and management for inventory management	Directly impacts Inventory HR/Mgmt, Training, Performance Assessment	[18, 31, 44, 49]	17
	Intermediary Vaccine Supply	Intermediate storage facility has vaccines to distribute	Directly impacts Facility Vaccine Supply	[31, 47, 60]	18
	Intermediary Storage Capacity	Intermediate storage facility has sufficient capacity in refrigerators and freezers to store vaccines	Moderates Intermediary Vaccine Supply	[31, 47, 60]	19
	Facility Distribution Method	Established means of transporting stock from intermediate storage to health facility	Directly impacts Facility Vaccine Supply	[31, 47, 60]	20
	Distribution Equipment	Vehicles and other equipment to distribute supply from intermediate storage to health facility	Moderates Facility Distribution Method	[31, 47, 60]	21
	Distribution HR/Mgmt	Adequate resources dedicated to distribution management	Moderates Facility Vaccine Supply, Intermediary Vaccine Supply	[31, 44, 52]	22
	Training	Adequate distribution management training	Moderates Distribution HR/Mgmt	[18, 27, 44, 50, 51–58]	23
	Performance Assessment	Procedures for distribution system performance assessment	Moderates Distribution HR/Mgmt	[11, 18, 54, 59, 60]	24
	Disbursement/Fund Mgmt	Adequate resources and management for distribution systems	Directly impacts Distribution HR/Mgmt, Training, Performance Assessment	[18, 31, 44, 49]	25
	Country Vaccine Supply	Central storage facility has vaccines to distribute	Directly impacts Intermediary Vaccine Supply	[18, 44, 50, 52, 65]	26
	Country Storage Capacity	Central storage facility has sufficient capacity in refrigerators and freezers to store vaccines	Moderates Country Vaccine Supply	[18, 44, 50, 52, 65]	27
	Intermediary Distribution Method	Established means of transporting stock from central to intermediate storage	Directly impacts Intermediary Vaccine Supply	[31, 47, 60]	28
	Distribution Equipment	Vehicles and other equipment to distribute supply from central to intermediate storage	Moderates Intermediary Distribution Method	[31, 47, 60]	29
	Leadership and Consistency	Oversight and decision-making in vaccine systems	Directly impacts Disbursement/Fund Mgmt, Outreach Protocol, Demand Generation Activities, Community Partnership, Service Cost, Supply LMIS	[8, 31, 44, 49, 66]	30
	Political Commitment	High-level support for vaccination systems	Directly impacts Leadership and Consistency, Funding	[11, 18, 37, 38, 49, 65, 67]	31
	Development Partner Participation	High-level engagement from development partners in vaccination systems	Directly Impacts Political Commitment, Funding, Country Vaccine Supply	[11, 18]	32
	Funding	Adequate high-level funding for vaccination systems	Directly impacts Disbursement/Fund Mgmt, Facility Infrastructure, Service Cost	[18, 31, 44, 49]	33
	Facility Readiness (Outreach)	Health facility is ready to vaccinate children through outreach activities	Directly impacts Utilization	[7, 9, 10, 18, 27, 31, 49, 51, 53]	34
	Outreach Transportation	Adequate vehicles to conduct outreach activities	Directly impacts Facility Readiness (Outreach)	[18, 31]	35
	Mobile Storage Capacity	Health facility has sufficient capacity to store vaccines during outreach activities	Directly impacts Facility Readiness (Outreach)	[31]	36
	Outreach Protocol	Procedures and plans for outreach activities	Directly impacts Mobile Storage Capacity, Outreach Transportation	[10, 60]	37
Decision to Vaccinate	Community Partnership	Active and positive relationship between health facility and community	Directly impacts Quality of Care, Community Awareness, Attitude	[10, 11, 27, 31, 37, 49, 56, 66, 67–70]	38
	Demand Generation Activities	Health facility encourages vaccine utilization in the community	Directly impacts Perceived Safety, Need, Effectiveness, Community Awareness, Attitude, Vaccine Awareness, Attitude	[8, 18, 31, 44, 56, 69, 71]	39
	Quality of Care	Quality of care at health facility for vaccination	Directly impacts Wait Time, Perceived Quality of Care	[30, 44, 50]	40
	Facility Infrastructure	Quality of health facility building and equipment	Directly impacts Wait Time, Perceived Quality of Care	[41, 44, 47, 53, 57]	41
	Service Cost	Cost of vaccine services charged to the mother or caretaker by the health facility	Moderates Ability to Finance Service	[8, 10, 30, 37, 41, 68, 72]	42
	Wait Time	Wait time at health facility for vaccination	Directly impacts Perceived Quality of Care	[8, 9, 27, 30, 45, 48, 54, 68, 72–75]	43
	Patient Volume	Number of patients who typically visit health facility	Directly impacts Wait Time	[9]	44
	Decision to Vaccinate	Mother or caretaker intends to vaccinate the child	Directly impacts Utilization	[8, 36, 41, 76]	45
	Vaccine Awareness, Attitude	Mother or caretaker is aware of vaccine and perceives it to be beneficial	Directly impacts Decision to Vaccinate, Perceived Eligibility	[8, 28–30, 32, 41, 46, 48, 49, 51, 53, 54, 56, 59, 61, 65, 69, 72–74, 77–83]	46
	Perceived Quality of Care	Mother or caretaker perceptions about the quality of care in the health facility	Directly impacts Vaccine Awareness, Attitude	[8, 9, 61, 83]	47
	Education	Mother or caretaker education	Directly impacts Vaccine Awareness, Attitude	[8–10, 32, 37, 39, 44, 48, 51, 57, 59, 61, 66, 75, 76, 78, 81–89]	48
	Prior Use	Child has been vaccinated previously	Directly impacts Vaccine Awareness, Attitude, Child Has Card	[8, 57, 66]	49
	Child Has Card	Child has vaccine card for recording doses	Directly impacts Vaccine Awareness, Attitude	[10, 32, 54, 72, 73, 79, 81]	50
	Perceived Safety, Need, Effectiveness	Mother or caretaker perceives that there is risk of disease and the vaccine is safe and effective	Directly impacts Vaccine Awareness, Attitude	[9, 29, 30, 38, 41, 45, 46, 51, 68, 72, 73, 75, 76–79, 90, 91]	51
	Community Awareness, Attitude	Influential community members are aware of the vaccine and perceive it to be beneficial	Directly impacts Vaccine Awareness, Attitude, Perceived Safety, Need, Effectiveness, Patient Volume	[10, 27, 37, 57, 71]	52
	Perceived Eligibility	Mother or caretaker perceives that child is eligible for vaccination	Directly impacts Decision to Vaccinate	[30, 57, 73, 77]	53
Child Eligibility	Child Eligibility	Child meets criteria to be eligible for vaccination	Directly impacts Utilization	[9, 48, 67, 70, 77, 78, 84, 92]	54
	Child Health	Child is healthy enough to be vaccinated	Directly impacts Child Eligibility, Perceived Eligibility	[7, 9, 29, 38, 45, 46, 48, 54, 57, 71, 72, 79, 90]	55
	Child Age	Child is of the appropriate age to be vaccinated	Directly impacts Child Eligibility, Perceived Eligibility	[9, 48, 67, 71, 77, 78, 84, 92]	56
	Child Sex	Sex of child	Directly impacts Perceived Eligibility	[9, 38, 39, 48, 51, 59, 61, 65, 67, 88]	57
Mother Able	Mother Able	Mother or caretaker has access to the means and time to vaccinate the child	Directly impacts Utilization	[9, 10, 41, 48, 50, 57, 75]	58
	Ability to Finance Service	Mother or caretaker can afford any cost of vaccine services	Directly impacts Mother Able, Decision to Vaccinate	[28, 45, 54, 68]	59
	Ability to Finance Transport	Mother or caretaker can afford any cost of transportation	Directly impacts Mother Able, Decision to Vaccinate	[10, 30, 41, 48, 68]	60
	Ability to Spare Time	Mother or caretaker can take time out of other responsibilities to vaccinate the child	Directly impacts Mother Able, Decision to Vaccinate	[9, 10, 28, 30, 46, 48, 57, 59, 61, 72, 73]	61
	Insurance	Whether the mother or caretaker has health insurance	Moderates Ability to Finance Service	[8, 9, 41, 75]	62
	SES	Socio-economic status of mother or caretaker	Directly impacts Ability to Finance Service	[10, 32, 37, 57, 67, 85, 88]	63
	Transport Time/Cost	Duration and cost of transportation to health facility incurred by mother or caretaker	Moderates Ability to Finance Transport, Ability to Spare Time	[10, 30, 41, 48, 68]	64
	Parity	Number of siblings of the child	Directly impacts Ability to Spare Time	[8–10, 29, 30, 39, 51, 57, 59, 61, 71, 75, 76, 79, 81, 83, 85, 88, 93]	65
	Distance	Distance from household to health facility	Directly impacts Transport Time/Cost	[10, 27, 30, 32, 39, 45, 48, 54, 56, 57, 61, 65, 79, 88, 93]	66
	Marital Status	Mother or caretaker marital status	Directly impacts Ability to Spare Time	[9, 39, 48, 57, 59, 75, 76, 78, 83, 87]	67
	Maternal Health	Mother or caretaker health	Directly impacts Mother Able, Decision to Vaccinate	[9, 45, 46, 48, 57]	68

**Table 3:** List of determinants to vaccine effectiveness

Theme	Determinant	Description	Relationship	Evidence	Label in Fig. 2
Vaccine Viability	Vaccine Viability	Vaccine is functional	Directly impacts Effectiveness	[12, 14, 55, 62, 94, 95]	69
	Cold Storage	Adequate storage temperature at each stage of the distribution system	Directly impacts Vaccine Viability	[55, 61–64, 95–109]	70
	Cold Transport	Adequate distribution temperature in distribution system	Directly impacts Cold Storage	[55, 61–64, 95–109]	71
	Monitoring	Systems in place for monitoring/correcting cold storage and transport	Directly impacts Cold Transport	[99, 103, 105, 109–113]	72
	Maintenance	Adequate resources dedicated to cold chain maintenance and repairs	Directly impacts Cold Storage, Cold Transport	[96, 99]	73
	Energy Source	Type of energy used to power cold storage	Directly impacts Cold Storage	[96, 99]	74
	Season/Weather	Seasonal and weather-related issues that may influence transportation and temperature	Directly impacts Cold Storage	[52, 57]	75
	HR	Adequate number and cadres of health facility staff	Directly impacts Coadministration, Cold Storage	[99, 102, 113–116]	76
	Training	Adequate health facility staff training	Moderates HR	[99, 102, 113–116]	77
	Vaccine Guidelines	Adequate guidelines for vaccine administration	Directly impacts Training	[99, 102, 117]	78
	Vaccine Formulation	Type of vaccine	Directly impacts Vaccine Guidelines	[12, 94, 99]	79
	Coadministration	Other vaccines administered to the child	Directly impacts Effectiveness	[118, 119]	80
Host Factors	Age at first dose	Age at which child received the first dose of the vaccine	Directly impacts Effectiveness	[12, 13, 67, 118, 120]	81
	Dosage Interval	Time between doses received	Directly impacts Effectiveness	[12, 14, 118, 120, 121]	82
	Acute Infection	Acute child health issues that may interfere with immune response	Directly impacts Effectiveness	[12, 13]	83
	Nutrition	Nutritional status of child	Directly impacts Effectiveness	[12, 13]	84
	Maternal Antibodies	Presence of maternal antibodies in child	Directly impacts Effectiveness	[12, 13]	85
	Breast Feeding	Breast feeding practices	Directly impacts Maternal Antibodies, Nutrition	[12, 13]	86
	Genetics	Genetic factors in child that may influence immune response	Directly impacts Effectiveness	[122]	87

### Synthesis of results

#### Thematic analysis

Thematic analysis was conducted concurrently with content analysis. The ways in which authors grouped together determinants were documented, and excerpts related to those themes were examined. This especially focused on the definition and justification of each theme according to the author. Authors’ descriptions regarding interactions between themes were also examined. Often, authors tended to use thematic groups as a convenient way to summarize their approach, reflecting their preconceptions about a theory of determinants. Of the 78 extracted articles, 41 relied on thematic groups.

**Fig. 3:**
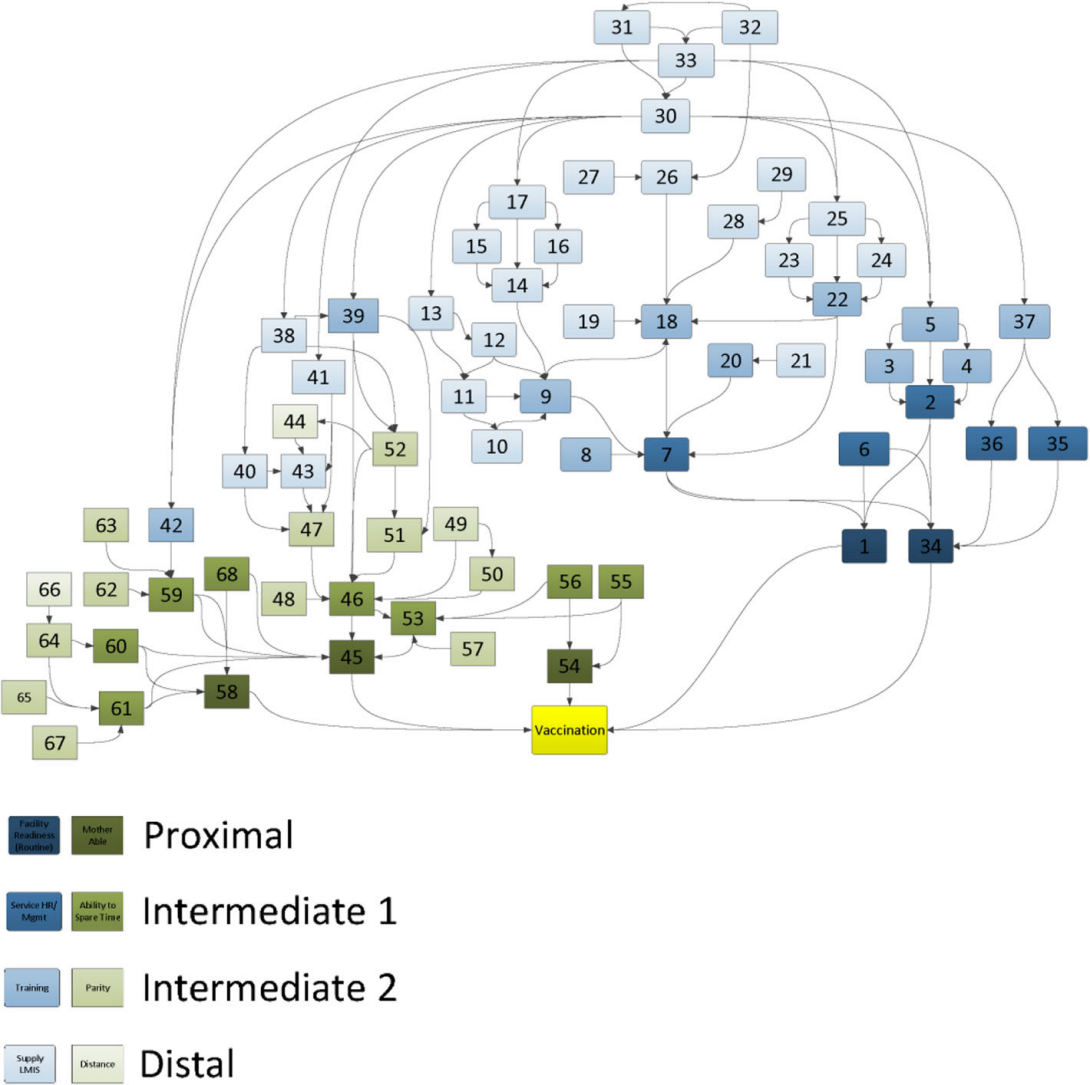
Network of determinants to vaccine effective coverage. This figure displays an example of findings from Thematic Analysis. Vaccination determinants in Fig. 2 have been labeled to represent one thematic model: proximity. For details on individual determinants, refer to Table 2

Three major thematic models (i.e. broader frameworks of themes) emerged from this analysis. One common model can be described as proximity. Authors who relied on proximity tended to group together determinants in terms of whether they were thought to directly impact vaccine coverage, or whether they were thought to impact vaccine coverage through a mediating factor. For example, Gauri and Kalenghian (2002) describe very high-level political, economic and social determinants in contrast to individual-level demand and acceptance, with accompanying description of the mechanisms of mediation [37]. Another common thematic model can be described as patient-centric. Numerous studies went into thorough detail conceptualizing the differences between determinants on the “demand side”, and tended to group all other determinants as “health system factors”. Chen (1986) for example provides a useful example of patient-centrism, in which a richness of information is provided about “biosocial” and “demand/utilization” factors, but health system factors are described with greater ambiguity, focusing on the high-level structure of the health system without any further detail [38]. In contrast, other models can be described as health system-centric, wherein more attention is given to factors on the “supply side”, and most other determinants are classified as “demand factors”. One example is Naimoli et al. (2008), who report four themes and 18 determinants relating to the health system, but only one theme with very little detail to describe all of demand [18]. Figure 3 displays the proximity model mapped to the content analysis.

Some areas of overlap emerged between themes. It was noted that the “health system factors” from patient-centric models, and the “demand factors” from health system-centric models often referred to the same thing: a more intermediate class of determinants that could be described as access or ability (both physical access and resource capacity). For example, Agot (2014) (one patient-centric study) emphasizes the health system, but describes health system factors as distance and cost incurred to the patient [39]. While these can be considered characteristics of the health system, they may usefully described as barriers between the child’s caretaker and the health workers. It was also noted that studies from high-income countries tended to describe a narrower range of determinants than studies from LMICs. Among these studies, few thematic groups pertained to the health system itself, with the most focusing on knowledge and perceptions and some pertaining to logistical limitations to access [8, 29].

#### Interpretive synthesis

Interpretive synthesis was the third analysis conducted, using the information from the systematic review. Interpretive synthesis followed the approach outlined by Dixon-Woods et al. (2006), [20] with the goal of generating a succinct and testable depiction of factors leading to vaccination. This entailed further comparison of themes, as well as integration of existing conceptual frameworks.

Three existing conceptual frameworks were explored in depth. The earliest framework found in the literature was described by Rudner-Lugo (1993) [8]. This model largely builds on the Health Belief Model [40], and describes perceived cost versus benefit of vaccination as the deciding determinant of vaccine utilization. The author depicts four demand-side factors and three supply-side factors contributing to that cost-benefit assessment. The second conceptual framework was put forward by Katz et al. (2010) [41]. These authors used systematic review and previous health behavior theories to generate what they title the Vaccine Perceptions, Accountability and Adherence Model. This model places additional emphasis on “cultural and economic forces”, while also recognizing the importance of barriers and “structural factors”. The third framework was proposed by LaFond et al. (2014) [11]. These authors offer a substantially different framework, focusing heavily on community engagement, awareness and commitment from high-level institutions such as government and development partners.

The conceptual framework we present draws lessons, similarities and differences from each of the above frameworks. The Rudner-Lugo framework lays out a useful structure of demand and supply-side factors coming together to lead to utilization, each with distinct contributing factors. The Katz et al. framework adds extended background to demand-side factors, especially with emphasis on perception-related decision making rather than strictly economic choices. The LaFond model brings an emphasis on community-level factors which was captured by neither of the other two. Our own thematic analysis (as discussed above) identified an important third construct between supply and demand: one relating to access and barriers to access. We therefore depict a framework based around three synthetic constructs: 1) health facility readiness to administer vaccines, 2) community-level access and 3) intention (on the part of the mother or caretaker) to vaccinate the child.

**Fig. 4:**
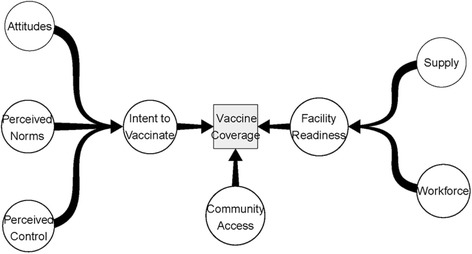
This figure displays the output from Interpretive Synthesis: a complete conceptual framework of vaccine determinants in its simplest possible form*.* Conceptual framework for vaccine coverage

General theories of health service utilization were used to describe contributors to the three primary constructs. Contributing factors for Health Facility Readiness were identified using the WHO Health System Building Blocks Framework [15]. This framework describes Supply of essential medicines and health Workforce as the most proximal components of a successful health system [15]. Contributing factors to Intent to Vaccinate were identified based on the Theory of Planned Behavior, a highly-cited behavior change model for health service utilization [36]. According to this theory, the three contributing factors to Intent are Attitudes, Perceived Norms, and Perceived Control, which is sometimes described as perceived self-efficacy. The resulting framework is depicted in Fig. 4.

The framework hypothesizes three principal determinants of vaccine utilization:

- Intent to Vaccinate - Demand for vaccines on the part of the mother that would result in vaccination in the absence of other barriers.
- Facility Readiness - Supply (by the health system) of vaccine services to adequately meet demand. Incorporates supplies (vials, syringes etc.), human resources and the consistency of their availability.
- Community Access - The ability (or inability) to successfully carry out the transaction of vaccine utilization, i.e. barriers and facilitators between Intent and Readiness.

Each principal determinant is also influenced by contributing factors, such as attitudes, norms and perceptions [36] for Intent to Vaccinate, and supply and workforce for Facility Readiness [15].

This framework represents the principal determinants in their most simplified form; as separate and distinct from one another. This was done in order to accomplish the main objective of producing a testable hypothesis, but does not preclude analysis of these constructs with correlation.

## Discussion

This study identifies three principal determinants of vaccine coverage: Intent to Vaccinate, Health Facility Readiness and Community Access. We present these results as part of the most comprehensive systematic review of vaccine determinants to date including three qualitative analyses and synthesis of the information we gathered into an evidence-based conceptual framework. The key advantages of the conceptual framework are that it is designed to be exhaustive, succinct and testable.

This systematic review has a key advantages over previous reviews. First, this review includes a broader set of studies than previous reviews. For example, Rudner Lugo (1993) [8] Falagas (2008) [9] Rainey (2011) [10] and LaFond (2014) [11] each performed systematic reviews to examine factors associated with vaccine coverage, but none of the reviews cited any of the others. Second, none of these studies examine the factors which impact the effectiveness of vaccines. On the contrary, Patriarca (1991) [12], Akande (2007) [13] and Cherry (2012) [14] explore reasons behind the effectiveness of vaccines but not utilization, and only focus on a subset of determinants. Beyond these, there are hundreds of studies which consider factors relating to either utilization or effectiveness but are not exhaustive in terms of the reasons they explore.

The conceptual framework presented here has advantages over previous frameworks as well. First, it represents a synthesis of multiple existing frameworks. Our analysis draws the most useful characteristics from each of them to avoid gaps that disadvantage other frameworks. Second, it was designed to be applicable in LMICs. Among the two previous systematic reviews which offer a conceptual framework [8, 11], only one of them focuses on LMICs, and that framework focuses on only a select few determinants [11]. Third, the conceptual framework presented here integrates the Theory of Planned Behavior and the WHO Building Blocks models. Both are vetted, tested models, the former of which has quantitatively outperformed other theories in direct comparisons [42]. Finally, this framework was designed to be quantitatively testable, a characteristic that is absent in at least some other frameworks. With appropriate data, the constructs in this framework could be directly represented using latent variable analysis. Future data collection and analysis plans could take advantage of this conceptual framework to be more grounded in existing research.

In light of this study’s strengths, a number of limitations remain. Although the network of determinants (Fig. 1) is intended to be comprehensive, one limitation of this study is completeness. There are four reasons why this study may not be considered complete. First, it is only complete within the context of the scientific literature. Any determinants that have been systematically overlooked by other researchers will not be present here. Second, the systematic review did not include studies of specific interventions to improve vaccine coverage, just research on determinants. Because of this, there is potential for our analyses to exclude potentially influential information. Third, it is only complete within a certain degree of proximity to utilization. One could continue to argue that each determinant has its own preceding determinants ad infinitum. While this may be true, it is clearly not the goal of this research to describe the entire spectrum of socioeconomic forces, thus only reasonably proximal determinants were described. Last, despite going to great lengths to uncover as many relevant articles as possible, it may be that some research studies were simply missed, or inaccurately assessed for relevance. Although the best practice for study completeness would have been a capture-recapture method, with a citation list of 1621 articles, we are confident that such studies are few [43]. Another potential limitation is that the entire study (web searches, review, analysis and synthesis) was conducted by a single researcher. While it would have been ideal to rely on multiple reviewers and assessment of inter-rater reliability, that was not feasible for this study.

## Conclusions

This study offers important contributions to the understanding of routine childhood vaccination in low and middle income countries. One is simply the list of studies it identified (Additional file 3). We consider this to be a useful public resource in its own right. Now that a near-complete list of studies exists, future researchers can perform their own qualitative analysis and develop competing conceptual frameworks with greater ease. Because of the results of the content analysis (Tables  1 and  2), researcher can easily look up other studies that discuss any determinant in particular. This study contributes conceptual framework of determinants of effective coverage of childhood vaccines that represents a synthesis of multiple existing frameworks, is applicable in low and middle-income countries, and is quantitatively testable. Future quantitative researchers can use this study to identify appropriate indicators to analyze and to define their theoretical model. By bringing together the collective evidence on the drivers of immunization, this review lends itself to better research in the future, further understanding of determinants, and greater progress against vaccine preventable diseases around the world.

## Additional files

Additional file 1: Protocol. (DOCX 21 kb)

Additional file 2: List of Potential Search Terms, Searches Conducted and Summary of Results. (XLSX 20 kb)

Additional file 3: Complete List of Citations, Sorted by Relevance. (XLSX 212 kb)

## Abbreviations

DALY: Disability-adjusted life year
DTP3: Diphtheria, tetanus, pertussis vaccine (3 doses)
LMICs: Low and middle-income countries
LOA: Lines-of-argument synthesis
RTA: Reciprocal translational analysis
WHO: World Health Organization
