# Supplementary material for: Determinants of effective vaccine coverage in low and middle-income countries: a systematic review and interpretive synthesis
Source: BMC Health Serv Res. 2017 Sep 26;17:681. doi: 10.1186/s12913-017-2626-0 (PMC5615444; doi:10.1186/s12913-017-2626-0)
Supplement: Supplementary file 1 — Protocol. (DOCX 21 kb) [file 12913_2017_2626_MOESM1_ESM.docx]

# Appendix A: Systematic Review Protocol

## Background

Routine childhood immunization is one of the most effective and cost-effective public health interventions. However, many children in low and middle-income countries (LMICs) remain unimmunized. Coverage with three doses of the diphtheria-tetanus-pertussis vaccine (DTP) ranges from 75% to 96%.(1) In addition to vaccine coverage, vaccine effectiveness stands as a major barrier to immunization. Effectiveness of measles vaccination for example may be as low as 66% in some LMICs.(2) In order to address the reasons for non-vaccination and vaccine failure in LMICs, they must first be understood.

Despite extensive effort on the part of major global health institutions including Gavi, the Vaccine Alliance, to understand the determinants of vaccination in LMICs, information gaps remain. Numerous research studies have been conducted to quantify and/or describe the major determinants and barriers to vaccination, but fewer have been conducted to understand determinants of effectiveness. Although some systematic reviews have been conducted to bring together studies on coverage determinants(3) or to bring together studies on effectiveness determinants(4), none, to our knowledge, have reviewed both coverage and effectiveness together, termed effective coverage. Furthermore, few studies have attempted to synthesize the broad determinants information found in the literature into a comprehensive, testable framework. To our knowledge, the only systematic reviews which have developed conceptual frameworks of determinants have focused solely on coverage (not effectiveness), and have been based on an incomplete subset of the whole body of literature on the topic.(5–7)

## Review Questions

This review seeks to establish, through the available literature, a comprehensive framework of determinants of effective coverage of vaccines, and to facilitate future studies on the same topic. The specific review questions to be addressed are:

1. What is the complete list of studies which contribute evidence about effective coverage determinants?
2. What is the complete list of determinants of effective coverage represented in the literature?
3. How, according to the literature, are effective coverage determinants hypothesized to relate to each other, and how are they hypothesized to lead to/impede immunization?

## Inclusion Criteria

### Types of studies

This review will include any observational, experimental, or quasi-experimental study that supplies quantitative or qualitative evidence supporting the existence of at least one determinant of effective coverage of childhood vaccines. It will be limited to English language studies only.

### Studies excluded

This review will exclude studies that do not include evidence related to childhood vaccines (e.g. adult vaccines or animal vaccines). It will exclude studies in which the primary goal was to a) assess the level or trend of vaccine coverage, b) assess the safety, efficacy, health impact or cost-effectiveness of vaccines or c) assess vaccine coverage in a highly-specific subpopulation (e.g. HIV-infected children in a particular community).

### Types of outcome measure

This review will include studies which seek determinants of vaccine coverage, vaccine effectiveness, or determinants of other determinants of immunization (e.g. determinants of maternal attitudes toward vaccination).

## Search Strategy

The search strategy will focus primarily on electronic databases:

1. Google Scholar
2. PubMed
3. Vaccine-specific and/or systematic review-specific databases (e.g. healthsystemsevidence.org)

An unstructured, preliminary review has already been conducted to assist the development of search terms. Search terms will be prioritized by expected novelty of results. Google scholar and PubMed search results terms will be systematically examined and every possibly-relevant search result will be compiled into a database. “Possibly-relevant” is defined as potentially meeting the inclusion criteria based on the title alone. Duplicate results will be tracked, and a stopping rule will be used to determine when no/few new articles are being discovered.

Once it has been determined that all possibly-relevant articles have been identified, vaccine-specific and systematic review-specific databases will be screened in their entirety to ensure completeness of the citation database. The references of any systematic review identified through this search process will be screened in their entirety.

Possibly-relevant studies that may meet inclusion criteria will be examined by their title and abstract for final determination about inclusion.

## Critical Appraisal

Identified studies that meet the inclusion criteria will be systematically assessed for their relevance to the study’s aims. Each study will be given a relevance score based on its comprehensiveness, emphasis, geography, novelty and outcomes explored. These categories will be assessed based on abstracts.

Data will be systematically extracted from the full text of a subset of articles in descending order of relevance. No pre-determined sample size for the subset of articles will be set, rather full text data extraction will continue until a subjective level of saturation has been reached, i.e. no new information is being gleaned from data extraction.

Extracted data will be compiled into a database, including geography, study design, study population, vaccine antigens included in the study, outcomes examined, the complete list of determinants supported by the study, major themes used to describe determinants, and any description of the relationships between determinants and immunization.

## Data Synthesis

Qualitative synthesis techniques will be used to formulate a conceptual framework from the extracted data and the content of the articles examined. We will follow guidelines of critical interpretive synthesis based on Dixon-Woods et al. (2006)(8) and thematic analysis based on May et al. (2001)(9)

A summary of the extracted data itself will also be provided to ensure transparency into the synthesis, and enable future researchers to formulate alternative conceptual frameworks.

## References

1. Harris JB, Gacic-Dobo M, Eggers R, Brown DW, Sodha SV, Control C for D, et al. Global routine vaccination coverage, 2013. MMWR Morb Mortal Wkly Rep. 2014;63(46):1055–8.

2. Cutts FT, Smith PG, Colombo S, Mann G, Ascherio A, Soares AC. Field evaluation of measles vaccine efficacy in Mozambique. Am J Epidemiol. 1990;131(2):349–355.

3. Falagas ME, Zarkadoulia E. Factors associated with suboptimal compliance to vaccinations in children in developed countries: a systematic review. Curr Med Res Opin. 2008;24(6):1719–1741.

4. Akande TM. A review of measles vaccine failure in developing countries. Niger Med Pract. 2007;52(5–6):112–6.

5. Rudner Lugo N. The Relationships among Service Delivery Factors, Community Characteristics, and Immunization Completion by Two-Year-Old Children Using County Health Departments [Internet]. 1993 [cited 2017 Jun 13]. Available from: http://elibrary.ru/item.asp?id=5765487

6. Rainey JJ, Watkins M, Ryman TK, Sandhu P, Bo A, Banerjee K. Reasons related to non-vaccination and under-vaccination of children in low and middle income countries: findings from a systematic review of the published literature, 1999–2009. Vaccine. 2011;29(46):8215–8221.

7. LaFond A, Kanagat N, Steinglass R, Fields R, Sequeira J, Mookherji S. Drivers of routine immunization coverage improvement in Africa: findings from district-level case studies. Health Policy Plan. 2014;czu011.

8. Dixon-Woods M, Cavers D, Agarwal S, Annandale E, Arthur A, Harvey J, et al. Conducting a critical interpretive synthesis of the literature on access to healthcare by vulnerable groups. BMC Med Res Methodol. 2006;6(1):35.

9. Mays N, Roberts E, Popay J. Synthesising research evidence. In: Studying the organisation and delivery of health services: research methods. London, UK: Routledge; 2001. p. 188–220.
